# Characterization of physical performance and change of direction deficit across age groups in young female volleyball players

**DOI:** 10.1186/s13102-025-01264-6

**Published:** 2025-07-19

**Authors:** João P Oliveira, Daniel A Marinho, Pedro Jacinto, Tatiana Sampaio, Jorge E. Morais

**Affiliations:** 1 University of Beira Interior, Covilhã, Portugal; 2 Research Centre for Active Living and Wellbeing (LiveWell), Instituto Politécnico de Bragança, Bragança, Portugal; 3 Research Center in Sports Sciences, Health Sciences and Human Development (CIDESD), Covilhã, Portugal; 4 Department of Sport Sciences, Instituto Politécnico de Bragança, Bragança, Portugal

**Keywords:** Volleyball, Young athletes, Neuromuscular performance, Change of direction, Agility testing

## Abstract

**Background:**

Volleyball performance relies heavily on agility, strength, and the ability to change direction, yet there is limited research investigating these physical attributes across different age groups in youth volleyball players. The change of direction (COD) deficit—a metric that isolates directional efficiency by comparing agility to linear speed—may provide a more nuanced understanding of an athlete’s movement capabilities. The aim of this study was to investigate: (i) the differences in anthropometrics, lower limb strength/power, dynamic balance, linear sprint speed, and change of direction (COD) ability between young volleyball age groups; (ii) the effect of age on COD deficit in young volleyball players, and; (iii) the COD deficit predictors.

**Methods:**

A cross-sectional analysis of young volleyball athletes was conducted. Seventy-seven female volleyball players from three age groups (U13:12.43 ± 0.89; U16:14.38 ± 0.52; U18:16.78 ± 0.93) participated in standardized tests measuring their physical qualities.

**Results:**

The older age groups (U18) were significantly taller, heavier, and faster in linear sprints than their younger counterparts. No significant differences between groups were observed in lower limb strength and power or dynamic balance. The U16 group demonstrated the lowest COD deficit. The regression analysis identified drop jump height (DJ) as the sole significant predictor of COD deficit, explaining 6.9% of the variance (R^2^ = 0.069, *p* = 0.033), highlighting its relevance as an indicator of reactive strength.

**Conclusions:**

These findings suggest that while anthropometric features have increased and sprint performance improved with age, there is no corresponding improvement in lower limb strength, power, or dynamic balance. Drop jump height was the sole predictor of COD deficit, emphasizing the importance of reactive strength in determining agility. Coaches should focus on enhancing lower limb strength and reactive power to optimize COD performance and prevent performance stagnation (training plateaus), particularly in older athletes.

## Background

Volleyball is a dynamic and fast-paced sport that demands a variety of rapid, multidirectional movements, including jumps, sprints, and quick changes of direction (COD) [1]. These movements are essential in offensive and defensive play, where athletes must respond quickly to the ball or opposing players [2]. Played on a relatively small court, volleyball requires explosive power, agility, and reactive strength to meet the demands of frequent transitions between attack and defense [3]. Success in volleyball is highly dependent on physical, technical, and tactical skills, with physical attributes like speed, power, and agility playing a fundamental role in performance [4]. These physical traits are frequently used as benchmarks for assessing talent potential and fitness in young volleyball athletes [5].

Volleyball players must sprint, decelerate, change direction, and jump with high frequency, all placing considerable stress on the lower limbs [2]. To better understand these physical demands, a variety of field-based tests have been developed to evaluate key physical abilities, including strength, power, speed, and dynamic balance [6]. These tests help profile an athlete’s physical condition and identify areas for improvement [5, 6]. In volleyball, COD performance is critical, as players frequently shift direction to block, spike, or defend against opposing attacks [7]. The T-agility test has been commonly used to evaluate COD abilities in volleyball players, offering valuable insight into their ability to change direction rapidly [8–10]. Additionally, tests like squat jumps (SJ), countermovement jumps (CMJ), and drop jumps (DJ) are widely used to assess lower limb strength and power, which are critical for executing explosive movements such as jumping and sprinting [6].

Dynamic balance is another critical factor in youth sports, particularly in injury prevention. The Y-balance test is one of the most reliable tools for assessing dynamic balance in young athletes, providing insights into their postural control during sports-specific movements [11]. Research has shown that athletes with poor dynamic balance are at an increased risk of injury [12], particularly in sports like volleyball, which involve frequent lateral movements and jumps [13, 14]. Given the demands of volleyball, maintaining dynamic balance is essential for executing these movements safely and effectively.

During adolescence, athletes undergo significant physical changes, particularly during growth spurts, which are associated with maturation and can lead to temporary reductions in coordination, balance, and strength [15]. As growth spurts occur, there is often disproportionate growth of the lower limbs compared to the upper body and trunk, which can negatively impact balance and motor coordination [16]. Research has suggested that these physiological changes can affect dynamic balance and COD performance, making it crucial to consider age and maturation when designing training programs for young athletes [16].

In addition to maturation, strength and power development play an essential role in the athletic performance of volleyball players, particularly during adolescence [17, 18]. Progressive overload, the systematic increase in training demands, is well-established as necessary for developing strength, power, and agility in young athletes [19]. However, some studies suggested that youth athletes may experience training plateaus if their programs do not evolve to match their growing physical capabilities, particularly in sports like volleyball, where explosive lower limb power is critical for performance [20–22]. Tests like the drop jump and countermovement jump are often used to assess an athlete’s ability to produce explosive power through the stretch-shortening cycle (SSC), a key factor in rapid movements such as jumping and sprinting [23, 24].

Although COD performance has been extensively studied in team sports like soccer and basketball, research in young volleyball players remains limited. In volleyball, athletes must execute frequent explosive movements in various directions, making COD ability crucial for both offensive and defensive actions [25]. Quick and reactive directional shifts are essential, especially during approach jumps, which are fundamental in executing offensive plays [9, 26]. To assess an athlete’s agility, the COD deficit—a metric that calculates the additional time needed to change direction compared to running a linear sprint over the same distance—has emerged as a valuable tool [27]. Recent approaches have proposed the use of the Change of Direction Deficit (COD deficit) as a more precise and isolated measure of an athlete’s ability to decelerate, reorient, and reaccelerate during multidirectional movements, rather than relying solely on raw COD performance times [28]. COD deficit is considered particularly relevant in youth athletic populations, where changes in physical attributes due to maturation may disproportionately affect sprint and agility metrics, potentially masking inefficiencies in change of direction ability [29]. This issue may be even more relevant in female volleyball, where match characteristics such as longer rallies, more complex defensive rotations, and frequent short-distance reorientations increase the importance of directional change efficiency [10]. Therefore, assessing COD deficit in this context may provide more meaningful insights into performance readiness and movement economy than conventional agility tests alone. Typically, older athletes, who benefit from greater physical development in terms of anthropometrics, strength, and power, demonstrate lower COD deficits [15, 30]. This relationship between physical maturation and COD deficit is particularly relevant in youth volleyball, where players’ physical abilities are still developing and evolving [30]. In Portugal, the Portuguese Volleyball Federation (FPV) classifies youth volleyball players into standardized categories based on chronological age [31]. These divisions are designed to structure competitive levels, ensuring players compete within age-appropriate cohorts. However, at the club level, team selection is often influenced by skill level, training needs, and competition demands, meaning that younger athletes may sometimes compete in older categories.

Given this variability in player placement, this study adopted a strict chronological age classification (U13, U16, U18) to ensure a consistent and homogeneous comparison of physical and performance attributes rather than basing groups on club-level team assignments. While previous studies have examined athletic performance in volleyball players, mainly at the elite level [25, 30, 32–35], a gap remains in understanding how key physical attributes—such as lower limb strength, dynamic balance, and COD ability—differ across younger volleyball age groups, particularly at lower competitive levels. Additionally, the influence of age on the COD deficit during this developmental period remains underexplored. Addressing these gaps is critical for developing age-appropriate training programs that can optimize performance and reduce injury risk. In applied settings, COD deficit may serve as a valuable tool for performance profiling and talent identification. Coaches can use this measure to detect athletes who, despite having high linear sprint speed, show limitations in their ability to efficiently execute directional changes—thus enabling the design of targeted training programs that emphasize deceleration control, directional technique, and asymmetry correction [29, 36].

Therefore, the aims of this study were to: (i) assess and compare a set of anthropometric, lower limbs’ strength and power, dynamic balance, linear sprints, COD test and COD deficit between young female volleyball age groups; (ii) analyze the effect of age on the COD deficit, and; (iii) identify the COD deficit predictors. It was hypothesized that older players would present bigger anthropometrics and better performances in all remaining variables, the COD deficit would improve with age, and this would be predicted by a mix of variables related to the players’ physical fitness.

## Methods

### Participants

The Portuguese Volleyball Federation (FPV) defines competitive age groups at the national level [31], similar to other international federations such as FIVB [37], CEV [38], and USA Volleyball [39], which also establish standardized youth divisions. However, despite these official classifications, club-level team placements often depend on age, skill level, training needs, and competition demands, leading to younger players competing in higher divisions. To ensure a homogeneous and objective comparison, this study categorized players strictly by chronological age (U13, U16, U18) rather than their club-assigned competitive squads, thus allowing for a clearer analysis of age-related performance differences.

The sample comprised 77 young female volleyball players recruited from one regional club. At the time of data collection, the players’ decimal age ranged from 10.51 to 18.21 years old (U13: 12.43 ± 0.89; U16: 14.38 ± 0.52; U18: 16.78 ± 0.93). They were divided into three groups according to their respective age squad (U13: *n* = 21, U16: *n* = 33, U18: *n* = 23). The players’ demographics are presented in Table 1. To ensure that the study had adequate statistical power, an a priori power analysis was conducted using G*Power [40]. The analysis indicated that a minimum of 66 participants was required to detect a large effect size (f² = 0.40) with 80% power (α = 0.05) for a one-way ANOVA (fixed effects, omnibus test). With a total sample of 77 participants, the study meets the necessary power requirements, ensuring reliable detection of meaningful differences between groups.

All three age groups had two weekly training blocks, each lasting between one and a half and two hours. They were evaluated immediately before their major regional competition and were considered Tier 2 athletes [41]. To be included in the measurements, players had to be completely free of pain at the time of the study and training regularly. If someone were receiving medical attention at the time or indicated any pain during the Y-balance test (please report to the methods section), they would be excluded from the study. Parents or guardians and players themselves signed an informed consent form. All procedures were by the Declaration of Helsinki regarding human research, and the Polytechnic Ethics Board approved the research (No. 127/2023).

### Research design

**Fig. 1:**
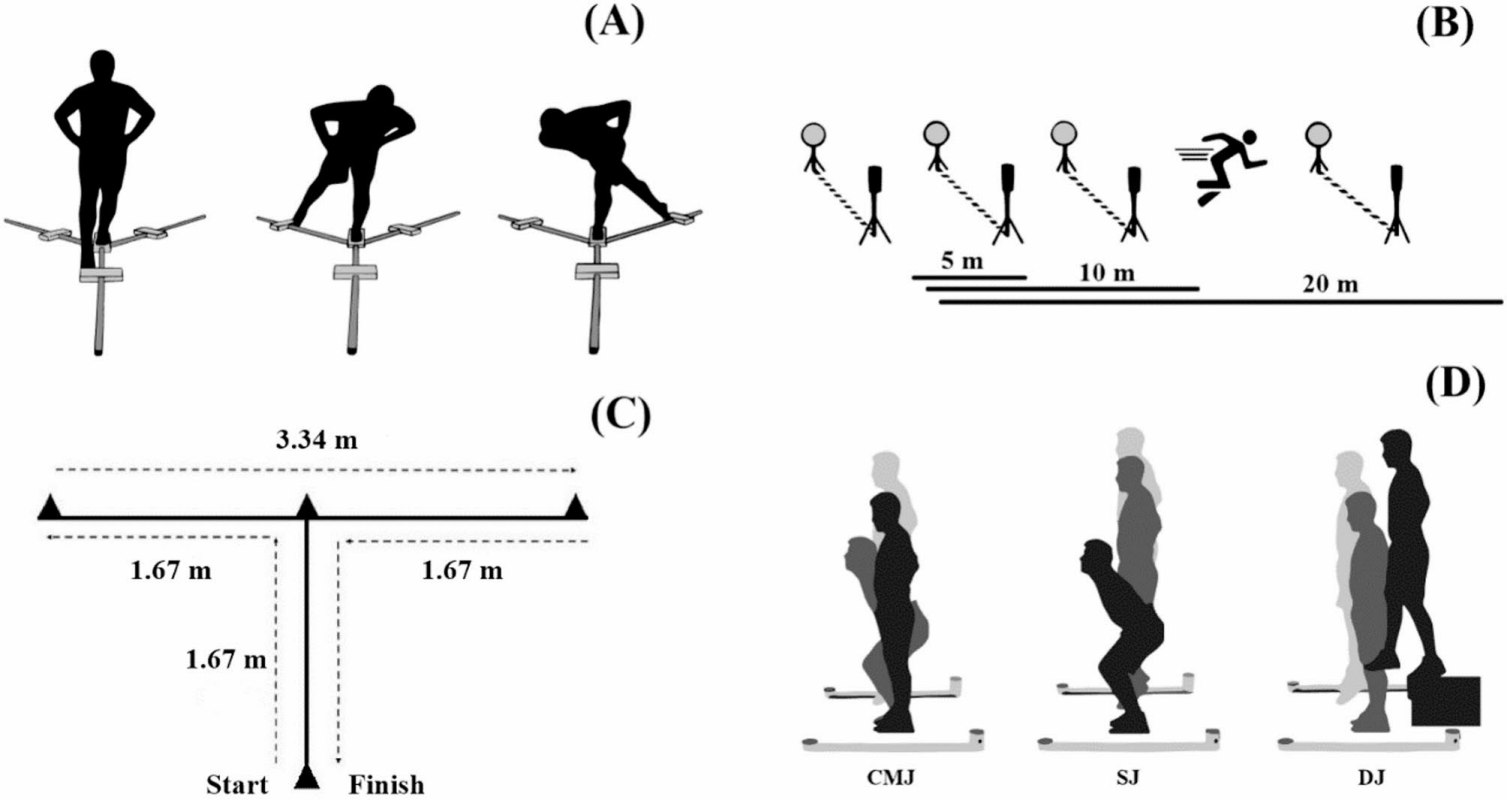
Visual presentation of the applied tests. Panel (**A**): Y-balance test; Panel (**B**): 20 m linear sprint test; Panel (**C**): 10 m agility T-test; Panel (**D**): jump tests (CMJ – countermovement jump; SJ – squat jump; DJ – drop jump)

This study characterized and compared a set of anthropometric, lower limbs’ strength and power, dynamic balance, linear sprints, COD test and COD deficit between young volleyball age groups. A cross-sectional analysis of young female volleyball athletes was conducted. The participants were split into three groups according to age (U13, U16 and U18). Figure 1 presents a visual illustration of the applied tests. The data collection took place on three separate days for each test, primarily due to logistical constraints and the high number of participants, ensuring that each test could be conducted under standardized conditions and with minimal fatigue. On the first day, anthropometric measurements and maturity offset calculations were performed. Lower limb strength and power tests were conducted on the second day, followed by dynamic balance assessments. The third day was dedicated to linear sprints and COD tests. Experienced sports scientists and coaches administered all tests. Each testing session was scheduled during their training sessions, and players were instructed to follow their regular diet and avoid strenuous activities the day before testing.

### Data collection

#### Anthropometrics and maturity offset

Body mass (in kg) was measured on an electronic scale (MC 780-P, Tanita, Tokyo, Japan) with minimal clothing. Height and sitting height (in cm) were measured using an electronic stadiometer (Seca 242, Seca, Hamburg, Germany). The maturity offset (MO, in years) and peak height velocity (PHV, in years) were calculated as suggested elsewhere [42]. The former represents the years an athlete is away from peak height velocity. If the offset is negative, the athlete has not yet reached peak height velocity. A positive offset indicates that the peak height velocity has already occurred.

#### Lower limbs’ strength and power

Before data collection, players performed a standardized warm-up based on muscle activation monitored by their coach. Afterward, they became familiar with the jumping tests’ protocols by performing each test with minimal effort to understand the biomechanics of each test and ensure the correct technique. The squat jump (SJ, in cm), countermovement jump (CMJ, in cm), and reactive strength index (RSI, in m/s) from the 45 cm drop jump (DJ, in cm) were used as indicators of lower limb strength and power [43]. A standardized 45 cm height was selected for the drop jump test based on practical feasibility given the large sample size and field-testing conditions, and in accordance with prior literature recommending this height as suitable for assessing reactive strength index in athletic populations [44]. Each player executed each test three times with a rest period of 30 s between the same jump and three minutes between different jump types to avoid any accumulated fatigue [45]. The best trial was used for further analysis [46]. All tests were measured with an Optojump system (Microgate, Bolzano, Italy) with the bars separated by 1 m. The validity and reliability of this equipment have already been confirmed [47]. Detailed protocols for each test can be found elsewhere [45, 46].

#### Dynamic balance of the lower trunk

The dynamic balance of the lower trunk was assessed using the Y-balance test [11]. The composite score (CS, in %) was generated by averaging and multiplying the sum of the three normalized reach distances by 100. Additionally, the absolute (in cm) and relative (in %) reach differences between lower limbs were calculated to evaluate reach symmetry. Deeper insights about this test can be consulted in other works [11, 48]. For qualitative analysis, it has been reported that CS’s less than 89% and symmetries greater than 4 cm are more likely to promote injuries [48]. Additional information regarding the Y-Balance protocol can be found elsewhere [46].

#### Linear sprint and change of direction tests

Before data collection, players performed a standardized warm-up based on muscle activation monitored by their coach. The linear sprint (partials taken at 5, 10 and 20-m) [49] and a modified version of the agility T-test (MAT) [50] were chosen as performance variables and collected on the same indoor court where they perform their training sessions and games. The agility T-test is considered a COD test based on the aspects of agility that need to be performed [51]. However, the generic cues in this test do not make the test well suited for sports such as volleyball because the total sprinting distance covered is approximately 40 m [50]. Therefore, we maintained the exact nature of displacement but reduced the total distance covered to 10 m (Fig. 1), to better replicate the distances players usually run during matches and to allow for calculations of the COD deficit using the 10 m sprint. Two sets of gates were placed at the starting and finish lines. This modified version of the T-Test has demonstrated high relative and absolute reliability, making it a consistent and sport-specific method for assessing agility in short-distance, multidirectional movements typical of court sports like volleyball [50]. Figure 1 shows the schematics of the two tests.

Participants performed a 20 m linear sprint test, which consisted of running this distance in a straight line in the shortest time. The players were encouraged to run at maximum speed for another 5 m after the 20-m mark to ensure that the distance was covered in the shortest possible time. Four sets of gates were placed at the starting line, 5-m, 10-m, and 20-m marks to retrieve information about each section. Participants performed each test three times at maximum speed, where players walked back to the starting line, actively resting. In contrast, the others performed the test, allowing for a rest period never less than 1–2 min between trials [49]. Subsequently, the fastest time was used for further analysis. All tests were timed with Microgate Witty photocells (Microgate, Bolzano, Italy). They were activated when crossed. The players were given directions to initiate their attempts 0.3-m before the first photocell and the timer started at their very first movement after crossing the photocells. They were advised to start whenever they felt ready to ensure a quicker and more consistent start [52]. The COD deficit was calculated as the time gap between the 10-m linear sprint time and the MAT test time as suggested by others [53, 54].

### Statistical analysis

All analyses were conducted using IBM SPSS software (Version 29; SPSS Inc., USA). The normality assumption was analyzed using the Shapiro–Wilk test, which revealed a normal distribution. The mean plus one standard deviation was calculated as descriptive statistics. The level of significance was set at α = 0.05. One-way ANOVA was used to analyze the differences between age groups (U13 vs. U16 vs. U18). Total eta square (η^2^) was selected as the effect size index, and interpreted as: (i) without effect if 0 < η^2^ < 0.04; (ii) minimum if 0.04 < η^2^ < 0.25; (iii) moderate if 0.25 < η^2^ < 0.64 and; (iv) strong if η^2^ > 0.64 [55]. Whenever appropriate, the Bonferroni correction was used to verify the differences between age groups (*p* < 0.017). Cohen’s d was used to estimate the standardized effect sizes, and interpreted as: (i) trivial if 0 ≤ d < 0.20; (ii) small if 0.20 ≤ d < 0.60; (iii) moderate if 0.60 ≤ d < 1.20; (iv) large if 1.20 ≤ d < 2.00; (v) very large if 2.00 ≤ d < 4.00; (vi) nearly distinct if d ≥ 4.00 [56]. Curve fitting was used to understand the relationship between COD deficit and age. The coefficient of determination (R^2^) was used to understand the magnitude of the relationship. Qualitatively, this was defined as: very weak if R^2^ < 0.04, weak if 0.04 ≤ R^2^ < 0.16, moderate if 0.16 ≤ R^2^ < 0.49, high if 0.49 ≤ R^2^ < 0.81, and very high if 0.81 ≤ R^2^ < 1.0. Simple linear regression (backward method) was used to test the COD deficit predictors and the R^2^ was used to understand the model variance.

## Results

Table 1 presents the descriptive statistics of players by age group. The U18 group showed the biggest anthropometric measures, followed by U16 and U13. However, this trend did not hold for lower limb strength and power, where U18 scored the highest, followed by U13 and U16. Similar scores were observed across all age groups in the upper and lower quarters regarding dynamic balance. All groups had lower quarter CS scores above the 89% cut-off, indicating a low risk of injury, except for the U16 group, which had a right limb CS score of 88.87%. In the upper quarter test, all groups scored 82–83%. In the 10 and 20 m linear sprints, U13 and U16 had similar times, while U18 was the fastest. In the T-agility test (COD), U18 was again the fastest, followed by U16 and U13. Notably, U16 showed the least COD deficit, followed by U18 and U13 (see Table 1).

**Table 1:** Descriptive statistics (mean and standard deviation – SD) of all variables by age-group. It is also presented the one-way ANOVA to identify the differences between groups

	U13 *n* = 21		U16 *n* = 33		U18 *n* = 23			
	**Mean**	**SD**	**Mean**	**SD**	**Mean**	**SD**	**F-ratio** (*p*-value)	**η** ^**2**^
	**Demographics and Anthropometrics**							
Decimal age [years]	12.43	0.89	14.38	0.52	16.78	0.93	179.70 (< 0.001)	0.83
Body mass [kg]	49.18	10.09	56.88	8.81	59.61	7.62	8.24 (< 0.001)	0.18
Height [cm]	158.41	6.98	163.69	4.90	167.10	5.48	11.98 (< 0.001)	0.26
Sitting height [cm]	79.85	3.31	84.17	2.74	85.95	3.56	20.24 (< 0.001)	0.37
Maturity offset [years]	0.74	0.89	2.23	0.51	3.80	0.85	86.149 (< 0.001)	0.72
PHV [years]	11.72	0.27	12.13	0.37	13.05	0.46	69.64 (< 0.001)	0.68
	**Strength and Power**							
CMJ height [cm]	24.86	6.50	24.28	4.31	27.26	5.92	1.90 (0.157)	0.06
SJ height [cm]	24.16	6.03	23.05	4.10	25.06	5.28	0.98 (0.379)	0.03
DJ height [cm]	22.46	5.67	22.07	4.41	23.52	4.79	5.49 (0.580)	0.02
Power [w/kg]	25.04	7.06	25.26	5.23	27.00	6.41	0.66 (0.520)	0.02
RSI [m/s]	0.76	0.31	0.77	0.23	0.84	0.29	0.61 (0.544)	0.02
	**Lower Quarter Dynamic Balance**							
CS_right_ [%]	90.57	8.42	88.87	5.89	90.35	6.83	0.50 (0.610)	0.01
CS_left_ [%]	90.91	8.85	90.39	6.39	91.75	6.59	0.24 (0.785)	0.01
	**Anterior differences**							
Absolute [cm]	1.91	1.71	3.17	2.57	3.03	2.95	2.45 (0.094)	0.06
Relative [%]	2.52	2.04	3.63	2.93	3.03	2.95	1.10 (0.339)	0.03
	**Posterolateral differences**							
Absolute [cm]	3.79	2.78	4.66	4.20	3.80	2.51	0.60 (0.550)	0.02
Relative [%]	4.85	3.92	5.39	5.02	4.52	2.96	0.304 (0.738)	0.01
	**Posteromedial differences**							
Absolute [cm]	4.95	2.96	4.33	3.46	3.76	1.97	0.90 (0.412)	0.02
Relative [%]	6.34	3.61	5.04	3.95	4.39	2.28	1.84 (0.167)	0.05
	**Upper Quarter Dynamic Balance**							
CS_right_ [%]	82.78	8.32	82.52	8.26	82.77	7.39	0.01 (0.992)	0.00
CS_left_ [%]	83.67	6.83	82.75	7.89	83.22	7.06	0.09 (0.915)	0.00
	**Medial differences**							
Absolute [cm]	4.29	4.58	3.20	3.44	3.42	2.25	0.55 (0.578)	0.02
Relative [%]	5.69	6.13	4.10	2.22	4.78	3.50	0.68 (0.511)	0.02
	**Superolateral differences**							
Absolute [cm]	3.58	4.41	3.86	3.39	4.40	4.15	0.22 (0.803)	0.01
Relative [%]	4.73	5.48	4.83	4.23	5.48	4.91	0.15 (0.862)	0.00
	**Inferolateral differences**							
Absolute [cm]	3.94	3.40	4.46	4.16	4.48	3.00	0.14 (0.873)	0.00
Relative [%]	5.29	4.41	5.41	5.16	5.43	3.42	0.01 (0.995)	0.00
	**Linear sprint**							
Time 10 m [s]	2.10	0.17	2.10	0.17	2.05	0.16	0.64 (0.532)	0.02
Time 20 m [s]	3.71	0.30	3.72	0.29	3.62	0.24	0.79 (0.458)	0.02
	**Agility**							
MAT [s]	4.31	0.51	4.20	0.42	4.19	0.27	0.54 (0.585)	0.02
COD deficit [s]	-2.20	0.47	-2.10	0.43	-2.14	0.40	0.37 (0.693)	0.01

PHV – peak height velocity; CMJ – countermovement jump; SJ – squat jump; DJ – drop jump; RSI – reactive strength index; CS – composite score; MAT – modified version of the agility T-test; COD – change of direction; η^2^ – eta square (effect size index)

Table 1 also presents the differences between groups. All anthropometric variables presented a significant group effect with a moderate to very high effect size. However, non-significant differences with very weak effect sizes were observed in all tests, indicating a very similar level of lower limbs strength and power, overall dynamic balance and also sprinting and agility abilities (Table 1). The same results were shown on the post-hoc corrections (Table 2), presenting the same trend. All anthropometric variables showed significant differences except for body mass, height and sitting height between the two older groups. Regarding strength and power, dynamic balance, linear sprints, and agility, non-significant differences were noted between age groups.

**Table 2:** Pairwise comparison of the variables between groups that presented significant differences (*p* < 0.017)

	U13 vs. U16				U13 vs. U18				U16 vs. U18			
	**Mean difference**	**95** **CI**	***p-value***	**d** **[descriptor]**	**Mean difference**	**95** **CI**	***p-value***	**d** **[descriptor]**	**Mean difference**	**95** **CI**	***p-value***	**d** **[descriptor]**
	**Demographics and Anthropometrics**											
Decimal age [years]	-1.956	-2.48 to -1.43	< 0.001	0.68 [moderate]	-4.348	-4.91 to -3.78	< 0.001	0.91 [moderate]	-2.393	-2.90 to -1.88	< 0.001	0.71 [moderate]
Body mass [kg]	-7.700	-13.75 to -1.65	0.008	9.32 [nearly distinct]	-10.437	-16.98 to -3.89	< 0.001	8.89 [nearly distinct]	N.S.			
Height [cm]	-5.290	-9.38 to -1.20	< 0.001	5.83 [nearly distinct]	-8.700	-13.09 to -4.30	< 0.001	6.26 [nearly distinct]	N.S.			
Sitting height [cm]	-4.322	-6.58 to -2.06	< 0.001	2.99 [very large]	-6.102	-8.53 to -3.67	< 0.001	3.44 [very large]	N.S.			
Maturity offset [years]	-1.487	-2.02 to -0.96	< 0.001	0.69 [moderate]	-3.054	-3.63 to -2.48	< 0.001	0.87 [moderate]	-1.567	-2.09 to -1.04	< 0.001	0.68 [moderate]
PHV [years]	-0.412	-0.68 to -0.14	< 0.001	0.33 [small]	-3.054	-3.63 to -2.48	< 0.001	0.38 [small]	-0.926	-1.19 to -0.66	< 0.001	0.41 [small]

PHV – peak height velocity; CMJ – countermovement jump; SJ – squat jump; DJ – drop jump; RSI – reactive strength index; CS – composite score; COD – change of direction; 95CI – 95% confidence intervals; d – Cohen’s d (effect size index); NS – non-significant

**Fig. 2:**
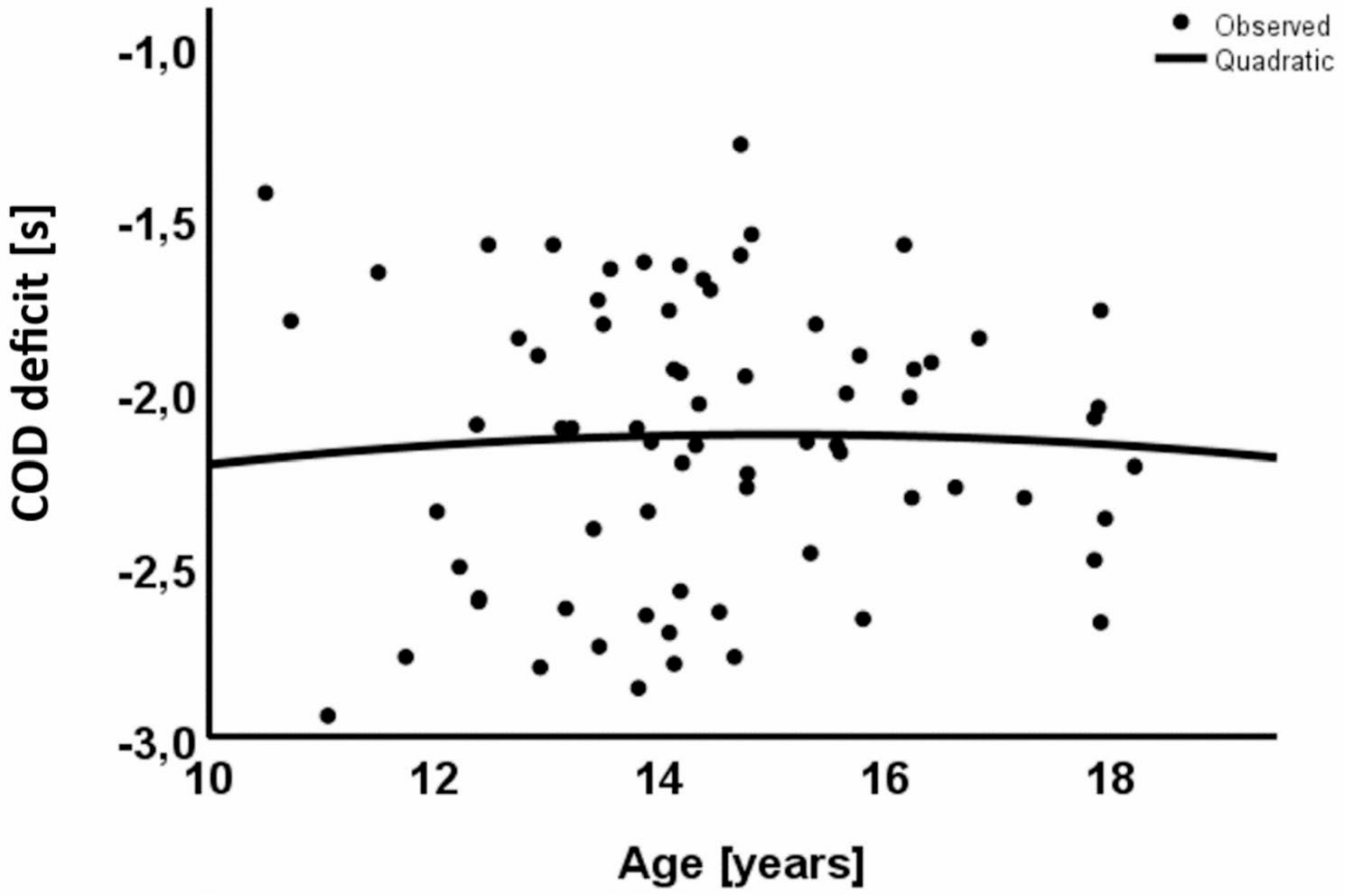
Linear regression between the COD deficit and age. COD – change of direction

Figure 2 shows the linear regression between COD deficit and age (R² = 0.001, *p* = 0.953), revealing no significant correlation between age and COD deficit in this population.

The regression analysis identified drop jump height (DJ) as the sole significant predictor of COD deficit, explaining 6.9% of the variance (R² = 0.069, β = -0.021, *p* = 0.033). Thus, the prediction equation is as follows:

$$\:COD\:deficit=-1.644 - 0.021 \bullet \:DJ$$

Where COD deficit is the change of direction deficit (in s), and DJ is the height obtained in the drop jump test (in cm).

## Discussion

The aims of this study were to: (i) assess and compare anthropometric measures, lower limb strength and power, dynamic balance, linear sprints, COD performance, and COD deficit across young female volleyball age groups; (ii) analyze the effect of age on the COD deficit; and (iii) identify the predictors of COD deficit in this population. The main findings revealed significant anthropometric differences between the age groups, with the U18 group showing bigger dimensions (body mass, height, and sitting height). However, lower limb strength and power did not show significant differences between groups, and dynamic balance remained consistent (no differences) across all ages. The U18 group demonstrated the fastest times in linear sprints and agility tests, while the U16 exhibited the smallest COD deficit. Additionally, body mass and posteromedial balance asymmetry were identified as key predictors of COD deficit in this population.

These findings partially support our initial hypothesis. Specifically, the U18 group exhibited significantly bigger anthropometric features and faster sprint and agility times than their counterparts but without significant differences in strength or power measures. The anticipated lower limb strength and dynamic balance improvements across age groups were not observed. The lack of significant differences in strength and balance between groups suggests that physical maturation alone may not lead to proportional improvements in these performance metrics. This contrasts with previous research on young female volleyball players, demonstrating a positive relationship between chronological age, anthropometric growth, lower limb power, and agility [35]. Differences in training exposure, competition level, and maturational variability may partially explain these discrepancies. It was noted that early-maturing players displayed significantly higher strength and power measures, suggesting that biological maturation influences performance outcomes [35]. Similarly, a meta-analysis across multiple sports emphasized that age-related gains in strength and power are commonly observed [16]. However, the absence of these improvements in our sample may indicate variations in training stimulus and differences in maturational timing within each age group.

Despite clear anthropometric progression, the absence of significant differences in lower limb strength and power raises meaningful considerations about the athletes’ training environments. In high-performance sports training, older athletes—particularly those nearing the end of adolescence—are typically expected to demonstrate better strength and power metrics due to natural maturation and more advanced training programs [14, 30, 32, 57]. Previous studies in volleyball have shown that structured strength and conditioning programs are critical for enhancing physical performance during periods of growth [7, 18, 20, 24, 32, 34, 57, 58]. However, the lack of differences in strength across these age groups suggests that the current training stimulus provided to these athletes may not adequately address the demands of volleyball, particularly in developing explosive power. It is important to note that this study did not assess the club’s training program. Therefore, while training factors may contribute to these results, they were not directly evaluated in this study. Future research should assess training exposure, load progression, and exercise selection to determine whether strength improvements are being effectively stimulated in volleyball players across different age groups. In contrast to other research where older athletes outperformed younger ones in strength and power due to progressive overload in training [35], our findings suggest that these athletes may not receive sufficient intensity or specificity in their conditioning programs. This highlights the need for coaches to reconsider the volume, intensity, and design of training stimuli to ensure that the strength adaptations align with the athletes’ physical growth and the demands of the sport.

Dynamic balance, assessed via the Y-balance test, showed consistent results across age groups. Still, the slightly lower scores observed in the U16 group raise concerns, particularly during periods of rapid growth. Previous research underscores the importance of balance training for youth athletes experiencing significant physical changes, as impaired dynamic balance has been linked to increased injury risks [59, 60]. With its frequent lateral movements and jumps, volleyball places high demands on dynamic balance [61, 62]. Previous studies have demonstrated that targeted neuromuscular control and balance training programs can improve these abilities [63–65], especially in youth populations [66–68]. Research has shown that growth spurts may cause temporary coordination deficits, as limb length changes can alter neuromuscular control [15, 60]. To better understand this, it is essential to consider how these growth-related changes affect an athlete’s ability to adjust body positioning and maintain stability during dynamic movements.

In the lower quarter Y-balance test, all groups scored above the 89% cut-off, indicating a low risk of injury in the lower limbs. However, the upper quarter scores were consistently below this threshold, signaling a higher risk of injury in the upper body. This finding is particularly significant given the demands of volleyball, which require rapid lateral and vertical movements. It is expected that dynamic balance may be influenced by maturational growth spurts, which are common during adolescence. These growth spurts often lead to temporary disruptions in coordination and balance due to disproportionate growth of the limbs relative to the trunk [15, 60]. During puberty, the lower limbs typically grow more rapidly, while the trunk and upper limbs lag slightly behind, causing a mismatch in body proportions [15, 60, 69]. This change affects the athlete’s center of mass, possibly explaining the weaker upper quarter balance scores across all groups. Additionally, growth spurts impact muscle strength, tendon stiffness, and neuromuscular control, further hindering flexibility and the body’s ability to adjust rapidly during dynamic movements [15, 16, 69].

Interestingly, the lower quarter balance scores remained similar across the groups despite the anticipated disruptions caused by growth [15, 16]. This may suggest that the current volleyball training programs, which emphasize lower body strength and agility, effectively address balance challenges. However, the consistently lower upper quarter scores suggest that upper body coordination may not receive enough attention in training. Volleyball-specific movements such as serving, spiking, and blocking, which rely on upper body control, likely demand a higher focus on fine motor coordination. This imbalance may increase the risk of injury and warrants attention in future training programs.

In addition to dynamic balance, COD abilities were a key focus of this study. The results from the MAT test, which was modified to reflect volleyball-specific movement patterns better and to match the 10-meter sprint distance necessary for COD deficit calculation, offered valuable insights into the athletes’ agility. The test’s combination of forward sprints, lateral shuffles, and backpedaling closely mirrors the movement patterns required during volleyball matches [50, 51]. This modification ensured that the agility test accurately replicated the athletes’ in-game actions, such as moving laterally to receive balls or adjusting the position for a block [2–4]. The COD deficit results showed that the U16 group had the lowest deficit, indicating superior COD abilities compared to U13 and U18. Despite expectations of continued improvement with age, the U18 group did not show a further reduction in COD deficit, which suggests a potential training plateau. This may reflect the need for more tailored and progressive training stimuli as athletes mature. Notably, the U16 age group may have benefited from a sensitive period of motor development and neuromuscular adaptation occurring around PHV, which can temporarily enhance agility and coordination [70]. As players progress beyond this phase, training must be adjusted to maintain improvements in agility and COD efficiency.

The regression analysis identified drop jump height (DJ) as the only significant predictor of COD deficit. Young et al., (2002) [71] compared the drop jump (DJ) test with eight different COD tests consisting of varying distances, turns, and straight sprints and suggested that the DJ test was significantly correlated with both straight sprinting speed and COD speed due to a similarity in the pushing-off actions [71]. This finding suggests that explosive lower limb power, as captured by the DJ, plays a crucial role in COD performance. The drop jump assesses an athlete’s reactive strength, or the ability to generate force upon landing rapidly and immediately re-accelerate [43, 72, 73]. This ability is essential for quick, forceful movements like those required in COD tasks in volleyball [7, 25]. Athletes who performed better in the DJ likely possess superior stretch-shortening cycle (SSC) efficiency, allowing them to minimize ground contact time during direction changes and improve their agility. Although both the DJ and CMJ assess SSC efficiency, DJ places a greater emphasis on reactive strength, making it more suited to measure the explosive demands of volleyball [2]. The CMJ, while useful in assessing lower limb power, measures SSC under more controlled conditions, which may not fully reflect the reactive, high-speed demands of volleyball-specific COD tasks. The DJ’s focus on quick transitions and minimal ground contact time may explain why it emerged as the more significant predictor in this study.

These findings reinforce the importance of incorporating plyometric training into volleyball-specific strength and conditioning programs. Given that explosive force application is critical for rapid changes of direction, volleyball coaches should consider implementing structured plyometric drills, such as depth jumps and bounding exercises, to enhance reactive strength and minimize COD deficits. The results of this study indicate a disconnect between the training stimulus and the physical demands of volleyball in this sample of players. Despite maturation, the lack of significant strength improvements suggests that training loads in this specific setting may not have progressed in a way that stimulates continued neuromuscular adaptation. Coaches working with athletes in comparable training environments may consider revisiting their strength and conditioning protocols, mainly focusing on progressive overload to further develop lower limb strength and power. By adjusting the training program’s volume, intensity, and specificity, it may be possible to optimize physical development for volleyball players under similar conditions. Given that DJ’s height was the only significant predictor of COD deficit, this study’s results indicate that improving reactive strength should be a key focus in volleyball training for players in this specific population. However, the absence of group differences in overall strength and power suggests that a well-balanced approach is necessary, incorporating explosive power development and progressive overload to optimize COD performance in young female volleyball players within this specific training context. Finally, the training plateau observed in the U18 group’s COD performance suggests that more advanced agility drills may be necessary for this sample of athletes. Coaches in similar competitive settings could consider introducing drills incorporating multidirectional movements, including diagonal and rotational actions, alongside reaction-based drills that mimic real-game scenarios. These adjustments may be beneficial in helping athletes facing similar performance plateaus to continue improving COD ability and overall volleyball performance.

## Limitations

As main limitations, it can be considered that: (i) this study exclusively examined female adolescent volleyball players from the same club. As a result, its applicability to male athletes remains unexplored. Given potential differences in physical development, training adaptations, and COD performance between male and female athletes, future research should investigate whether similar trends and predictors of COD deficit apply to male volleyball players; (ii) since all participants were recruited from a single club, the findings may not fully represent the variability present in volleyball players from different teams, competitive levels, or training environments. Future research should include athletes from multiple clubs and leagues to enhance the generalization of the results. Indeed, since all players were from the same club and followed similar training programs, one can argue that the younger athletes may evolve similarly to the older ones. Therefore, longitudinal studies might provide deeper insights to understand these trends better and provide a more accurate overview of how training and maturation influence young volleyball players’ performance; (iii) lifestyle factors such as nutrition, sleep, and recovery strategies were not controlled. Future research should consider incorporating these variables to gain a more comprehensive understanding of the external factors influencing COD performance in young volleyball players. (iv) the standardized use of a fixed 45 cm drop jump height for all participants. Although this approach was selected based on practical feasibility and supported by the recommendations, individualized drop heights might provide higher sensitivity in RSI assessment. Future studies should explore individualized heights where possible.

## Conclusions

Older volleyball players were taller, heavier, and faster in linear sprints. However, no significant differences in lower limb strength and power were observed across age groups, suggesting that physical maturation did not translate into the expected performance gains. Interestingly, the upper quarter dynamic balance was consistently weaker across all groups, possibly related to coordination challenges during growth spurts. The COD deficit was lowest in the U16 group but did not improve further in the U18 group, potentially indicating a training plateau. In this population, DJ’s height was the sole predictor of COD deficit, highlighting the importance of reactive strength for agility. However, it is important to note that this only explained 6.9% of the variance in COD deficit, indicating limited predictive power. This suggests that other unaccounted factors, such as neuromuscular control, movement strategy, or sport-specific training adaptations, may also play a role in COD performance. Future research should explore additional predictive variables to provide a more comprehensive understanding of the determinants of COD ability in volleyball players. Coaches operating in similar training contexts should prioritize progressive overload and incorporate advanced agility drills to address training plateaus in older athletes. Moreover, specific attention to upper body coordination could help reduce injury risks and enhance overall performance as athletes mature. While the current findings offer practical insights for training optimization, the limited explanatory power of the model highlights the need for individualized training approaches that consider multiple contributing factors beyond reactive strength alone.

## Data Availability

The datasets generated and/or analyzed during the current study are not publicly available due to privacy reasons but are available from the corresponding author on reasonable request.

## Abbreviations

U13: Under 13
U16: Under 16
U18: Under 18
COD: Change of direction
CMJ: Countermovement jump
SJ: Squat jump
DJ: Drop jump
CS: Composite score
PHV: Peak height velocity
RSI: Reactive strength index
NS: Non-significant
CI: Confidence intervals
FPV: Portuguese volleyball federation
FIVB: Fédération internationale de volleyball (international volleyball federation)
CEV: Confédération Européenne de volleyball (european volleyball confederation)
USA Volleyball: United states volleyball federation

## References

[1] Silva AF, Clemente FM, Lima R, Nikolaidis PT, Rosemann T, Knechtle B. The effect of plyometric training in volleyball players: A systematic review. Int J Environ Res Public Health. 2019;16:2960. 31426481 10.3390/ijerph16162960 PMC6720263

[2] Lidor R, Ziv G. Physical and physiological attributes of female volleyball players-a review. J Strength Conditioning Res. 2010;24:1963–73. 10.1519/JSC.0b013e3181ddf835 20543736

[3] Ho C-S, Lin K-C, Chen K-C, Chiu P-K, Chen H-J. System design and application for evaluation of blocking agility in volleyball. Proc Institution Mech Eng Part P: J Sports Eng Technol. 2016;230:195–202.

[4] Farrow D, Young W, Bruce L. The development of a test of reactive agility for netball: a new methodology. J Sci Med Sport. 2005;8:52–60. 15887901 10.1016/s1440-2440(05)80024-6

[5] Ntozis C, Cherouveim E, Gountas K, Bakodimos G, Apostolidis N, Tsolakis C. Relative age effect in Greek female young volleyball players: data from the National talent identification program. J Phys Educ Sport. 2021;21:1967–75.

[6] Wright WC. The efficacy of performance tests and testing instruments within elite female volleyball players. The University of Alabama 2023.

[7] Lockie RG, Dawes JJ, Callaghan SJ. Lower-body power, linear speed, and change-of-direction speed in division I collegiate women’s volleyball players. Biology Sport. 2020;37:423–8. 10.5114/biolsport.2020.96944 PMC7725048 33343076

[8] Fry AC, Kraemer WJ, Weseman CA, Conroy BP, Gordon SE, Hoffman JR, et al. The effects of an off-season strength and conditioning program on starters and non-starters in women’s intercollegiate volleyball. J Strength Conditioning Res. 1991;5:174–81.

[9] Chuang C-H, Hung M-H, Chang C-Y, Wang Y-Y, Lin K-C. Effects of agility training on skill-related physical capabilities in young volleyball players. Appl Sci. 2022;12:1904.

[10] Gabbett T, Georgieff B. Physiological and anthropometric characteristics of Australian junior national, state, and novice volleyball players. J Strength Conditioning Res. 2007;21:902–8. 10.1519/R-20616.1 17685708

[11] Plisky PJ, Gorman PP, Butler RJ, Kiesel KB, Underwood FB, Elkins B. The reliability of an instrumented device for measuring components of the star excursion balance test. North Am J Sports Phys Therapy: NAJSPT. 2009;4:92. PMC2953327 21509114

[12] Butler RJ, Lehr ME, Fink ML, Kiesel KB, Plisky PJ. Dynamic balance performance and noncontact lower extremity injury in college football players: an initial study. Sports Health: Multidisciplinary Approach. 2013;5:417–22. 10.1177/1941738113498703 PMC3752196 24427412

[13] Polglaze T, Dawson B, Peeling P. Gold standard or fool’s gold?? The efficacy of displacement variables as indicators of energy expenditure in team sports. Sports Med. 2016;46:657–70. 26643522 10.1007/s40279-015-0449-x

[14] Sheppard JM, Gabbett TJ, Stanganelli L-CR. An analysis of playing positions in elite men’s volleyball: considerations for competition demands and physiologic characteristics. J Strength Conditioning Res. 2009;23:1858–66. 10.1519/JSC.0b013e3181b45c6a 19675472

[15] Malina RM. Growth, maturation, and physical activity. Hum Kinetics. 2004.

[16] Albaladejo-Saura M, Vaquero-Cristóbal R, González-Gálvez N, Esparza-Ros F. Relationship between biological maturation, physical fitness, and kinanthropometric variables of young athletes: A systematic review and meta-analysis. Int J Environ Res Public Health. 2021;18:328. 33466291 10.3390/ijerph18010328 PMC7795393

[17] Fattahi A, Sadeghi H. Resistance, plyometrics and combined training in children and adolescents’ volleyball players: A review study. J Sci Res Rep. 2014;3:2584–610.

[18] Kitamura K, Roschel H, Loturco I, Lamas L, Tricoli V, João PV, et al. Strength and power training improve skill performance in volleyball players. Motriz: Revista De Educação Física. 2020;26:e10200034.

[19] Drury B, Ratel S, Clark CC, Fernandes JF, Moran J, Behm DG. Eccentric resistance training in youth: perspectives for long-term athletic development. J Funct Morphology Kinesiol. 2019;4:70. 10.3390/jfmk4040070 PMC7739302 33467385

[20] Kraemer WJ, Caldwell LK, Barnhart EC. Developing a resistance training program for volleyball. In: Reeser JC, Bahr R, editors. Handbook of Sports Medicine and Science. 1st edition. Wiley 2017;38–48.

[21] Stratton G, Oliver JL. The impact of growth and maturation on physical performance. Strength and conditioning for young athletes. Routledge 2019;3–20.

[22] Lloyd RS, Oliver JL, Faigenbaum AD, Myer GD, Croix MBDS. Chronological age vs. biological maturation: implications for exercise programming in youth. J Strength Conditioning Res. 2014;28:1454–64. 10.1519/JSC.0000000000000391 24476778

[23] Markovic G, Dizdar D, Jukic I, Cardinale M. Reliability and factorial validity of squat and countermovement jump tests. J Strength Conditioning Res. 2004;18:551–5. 10.1519/1533-4287(2004)18<551:RAFVOS>2.0.CO;2 15320660

[24] Sheppard J, Hobson S, Barker M, Taylor K, Chapman D, McGuigan M, et al. The effect of training with accentuated eccentric load Counter-Movement jumps on strength and power characteristics of High-Performance volleyball players. Int J Sports Sci Coaching. 2008;3:355–63.

[25] Hedrick A. Training for high level performance in women’s collegiate volleyball: part I training requirements. Strength Conditioning J. 2007;29:50–3.

[26] Forthomme B, Croisier J-L, Ciccarone G, Crielaard J-M, Cloes M. Factors correlated with volleyball Spike velocity. Am J Sports Med. 2005;33:1513–9. 16009986 10.1177/0363546505274935

[27] Pleša J, Kozinc Ž, Šarabon N. The association between force-velocity relationship in countermovement jump and sprint with approach jump, linear acceleration and change of direction ability in volleyball players. Front Physiol. 2021;12:763711. 34867467 10.3389/fphys.2021.763711 PMC8637321

[28] Nimphius S, Callaghan SJ, Spiteri T, Lockie RG. Change of direction deficit: A more isolated measure of change of direction performance than total 505 time. J Strength Conditioning Res. 2016;30:3024–32. 10.1519/JSC.0000000000001421 26982972

[29] Dos’ Santos T, Thomas C, Comfort P, Jones PA. Comparison of change of direction speed performance and asymmetries between team-sport athletes: application of change of direction deficit. Sports. 2018;6:174. 30545155 10.3390/sports6040174 PMC6315619

[30] Koźlenia D, Popowczak M, Horička P, Šimonek J, Domaradzki J. Sex-specific relationship patterns between body morphology and maturity status with change of direction and agility in elite adolescent volleyball players. Sci Rep. 2024;14:13170. 38849450 10.1038/s41598-024-64190-6 PMC11161477

[31] Federação. Portuguesa de Voleibol. Circular N^o^ 21–2010/2011.

[32] Marques MC, Van Den Tillaar R, Vescovi JD, González-Badillo JJ. Changes in strength and power performance in elite senior female professional volleyball players during the in-season: a case study. J Strength Conditioning Res. 2008;22:1147–55. 10.1519/JSC.0b013e31816a42d0 18545195

[33] Mielgo-Ayuso J, Calleja-González J, Clemente-Suárez VJ, Zourdos MC. Influence of anthropometric profile on physical performance in elite female volleyballers in relation to playing position. Nutr Hosp. 2015;31:849–57. 10.3305/nh.2015.31.2.7658 25617573

[34] Uslu S, Abazović E, Čaušević D, Mahmutović I, Riza B. The relationship between isokinetic strength and jump performance in elite female volleyball players. Acta Kinesiologica. 2021;1.

[35] Albaladejo-Saura M, Vaquero-Cristóbal R, García-Roca JA, Esparza-Ros F. Influence of maturity status on kinanthropometric and physical fitness variables in adolescent female volleyball players. Appl Sci. 2022;12:4400. 10.7717/peerj.13216 PMC8992641 35402095

[36] Lloyd RS, Oliver JL, Faigenbaum AD, Howard R, Croix MBDS, Williams CA, et al. Long-term athletic development, part 2: barriers to success and potential solutions. J Strength Conditioning Res. 2015;29:1451–64. 10.1519/01.JSC.0000465424.75389.56 25909962

[37] Fédération Internationale de Volleyball (FIVB). Age category competitions in volleyball and beach volleyball. 2023.

[38] European Volleyball. Confederation (CEV). Age-group championships. 2023.

[39] USA Volleyball (USAV). 2024-25 Junior age divisions. 2024.

[40] Faul F, Erdfelder E, Buchner A, Lang A-G. Statistical power analyses using G* power 3.1: tests for correlation and regression analyses. Behav Res Methods. 2009;41:1149–60. 19897823 10.3758/BRM.41.4.1149

[41] McKay A, Stellingwerff T, Smith E, Martin D, Mujika I, Goosey-Tolfrey V, et al. Defining training and performance caliber: A participant classification framework. Int J Sports Physiol Perform. 2022;17:317–31. 34965513 10.1123/ijspp.2021-0451

[42] Kozieł SM, Malina RM. Modified maturity offset prediction equations: validation in independent longitudinal samples of boys and girls. Sports Med. 2018;48:221–36. 28608181 10.1007/s40279-017-0750-y PMC5752743

[43] Verkhoshansky Y, Verkhoshansky N. Special strength training: manual for coaches. Verkhoshansky Sstm Rome; 2011.

[44] Newton RU, Dugan E. Application of strength diagnosis. Strength Conditioning J. 2002;24:50–9.

[45] Healy R, Kenny IC, Harrison AJ. Assessing reactive strength measures in jumping and hopping using the Optojump^™^ system. J Hum Kinetics. 2016;54:23–32. 10.1515/hukin-2016-0032 PMC5187958 28031754

[46] Sampaio T, Marinho D, Teixeira JE, Oliveira J, Morais J, Clustering. U-14 Portuguese regional team football players by lower limb strength, power, dynamic balance, speed and change of direction: Understanding the field position factor. PeerJ. 2023;11:e15609. 37483964 10.7717/peerj.15609 PMC10362840

[47] Glatthorn JF, Gouge S, Nussbaumer S, Stauffacher S, Impellizzeri FM, Maffiuletti NA. Validity and reliability of Optojump photoelectric cells for estimating vertical jump height. J Strength Conditioning Res. 2011;25:556–60. 10.1519/JSC.0b013e3181ccb18d 20647944

[48] Butler RJ, Lehr ME, Fink ML, Kiesel KB, Plisky PJ. Dynamic balance performance and noncontact lower extremity injury in college football players: an initial study. Sports Health. 2013;5:417–22. 24427412 10.1177/1941738113498703 PMC3752196

[49] Naser N, Ali A. A descriptive-comparative study of performance characteristics in futsal players of different levels. J Sports Sci. 2016;34:1707–15. 26800448 10.1080/02640414.2015.1134806

[50] Sassi RH, Dardouri W, Yahmed MH, Gmada N, Mahfoudhi ME, Gharbi Z. Relative and absolute reliability of a modified agility T-test and its relationship with vertical jump and straight sprint. J Strength Conditioning Res. 2009;23:1644–51. 10.1519/JSC.0b013e3181b425d2 19675502

[51] Jones PA, Nimphius S. Change of direction and agility. Performance assessment in strength and conditioning. Routledge 2018;140–65.

[52] Nuell S, Illera-Domínguez VR, Carmona G, Alomar X, Padullés JM, Lloret M, et al. Hypertrophic muscle changes and sprint performance enhancement during a sprint-based training macrocycle in national-level sprinters. Eur J Sport Sci. 2020;20:793–802. 31526116 10.1080/17461391.2019.1668063

[53] Loturco I, Pereira LA, Reis VP, Abad CC, Freitas TT, Azevedo PH, et al. Change of direction performance in elite players from different team sports. J Strength Conditioning Res. 2022;36:862–6. 10.1519/JSC.0000000000003502 32168177

[54] Little T, Williams AG. Specificity of acceleration, maximum speed, and agility in professional soccer players. J Strength Conditioning Res. 2005;19:76–8. 10.1519/14253.1 15705049

[55] Ferguson CJ. An effect size primer: A guide for clinicians and researchers. Prof Psychology: Res Pract. 2009;40:532–8.

[56] Hopkins W. A scale of magnitudes for effect statistics. A new view of statistics. 2002. http://sportsci.org/resource/stats/effectmag.html html (10 October 2013). 2019.

[57] Makaruk H, Sacewicz T. Effects of plyometric training on maximal power output and jumping ability. Hum Mov. 2010;11:17–22.

[58] Freitas TT, Pereira LA, Alcaraz PE, Arruda AFS, Guerriero A, Azevedo PHSM, et al. Influence of strength and power capacity on change of direction speed and deficit in elite Team-Sport athletes. J Hum Kinetics. 2019;68:167–76. 10.2478/hukin-2019-0069 PMC6724583 31531142

[59] Meyers RW, Oliver JL, Hughes MG, Lloyd RS, Cronin JB. New insights into the development of maximal sprint speed in male youth. Strength Conditioning J. 2017;39:2–10.

[60] Philippaerts RM, Vaeyens R, Janssens M, Van Renterghem B, Matthys D, Craen R, et al. The relationship between peak height velocity and physical performance in youth soccer players. J Sports Sci. 2006;24:221–30. 16368632 10.1080/02640410500189371

[61] Polglaze T, Dawson B. The physiological requirements of the positions in state league volleyball. Sports Coach. 1992;15:32–32.

[62] Zhang Z, Jiang M, Jing Y, Li M, Li Y, Yang X. Associations between sprint mechanical properties and change of direction ability and asymmetries in COD speed performance in basketball and volleyball players. Life. 2024;14:1434. 39598231 10.3390/life14111434 PMC11595913

[63] Fuchs PX, Fusco A, Cortis C, Wagner H. Effects of differential jump training on balance performance in female volleyball players. Appl Sci. 2020;10:5921.

[64] Zarei M, Soltani Z, Hosseinzadeh M. Effect of a proprioceptive balance board training program on functional and neuromotor performance in volleyball players predisposed to musculoskeletal injuries. Sport Sci Health. 2022;18:975–82.

[65] Guzmán-Muoz E, Rodríguez SS, Concha-Cisternas Y, Badilla P, Méndez-Rebolledo G. The effects of neuromuscular training on the postural control of university volleyball players with functional ankle instability: a pilot study. Arch De Med Del Deporte. 2020;36:283–7.

[66] Turgut E, Colakoglu FF, Serbes P, Akarcesme C, Baltacı G. Effects of 12-week in-season low-intensity plyometric training on dynamic balance of pre-pubertal female volleyball players. Turkish J Sport Exerc. 2017;19:24–30.

[67] Pau M, Loi A, Pezzotta MC. Does sensorimotor training improve the static balance of young volleyball players? Sports Biomech. 2012;11:97–107. 22518948 10.1080/14763141.2011.637126

[68] Trajković N, Bogataj Š. Effects of neuromuscular training on motor competence and physical performance in young female volleyball players. Int J Environ Res Public Health. 2020;17:1755. 32182680 10.3390/ijerph17051755 PMC7084803

[69] Beunen GP, Malina RM, Van’t Hof MA, Simons J, Ostyn M, Renson R, et al. Adolescent growth and motor performance: A longitudinal study of Belgian boys. Human Kinetics 1988.

[70] Lloyd RS, Oliver JL. The youth physical development model: A new approach to long-term athletic development. Strength Conditioning J. 2012;34:61–72.

[71] Young WB, James R, Montgomery I. Is muscle power related to running speed with changes of direction? J Sports Med Phys Fitness. 2002;42:282–8. 12094116

[72] Pedley JS, Lloyd RS, Read P, Moore IS, Oliver JL. Drop jump: A technical model for scientific application. Strength Conditioning J. 2017;39:36.

[73] Pleša J, Kozinc Ž, Šarabon N. Bilateral deficit in countermovement jump and its influence on linear sprinting, jumping, and change of direction ability in volleyball players. Front Physiol. 2022;13:768906. 35185609 10.3389/fphys.2022.768906 PMC8847223

